# The Nature of Associations between Physical Stimulus Size and Left-Right Response Codes

**DOI:** 10.5334/joc.206

**Published:** 2022-02-01

**Authors:** Melanie Richter, Peter Wühr

**Affiliations:** 1 TU Dortmund, Dortmund, DE

**Keywords:** correspondence effect, physical stimulus size, response location, polarity correspondence principle, continuous, categorical

## Abstract

In two-choice response tasks, participants respond faster and more accurate with the left hand to a small stimulus and with the right hand to a large stimulus as compared to the reverse assignment. This compatibility effect suggests the existence of associations between cognitive codes of physical stimulus size and cognitive codes of left/right responses. Here, we explore the nature of associations between stimulus-size codes and left/right response codes by using more levels of stimulus size than in our previous studies. For example, the strengths of the associations between stimulus-size codes and response codes might either change gradually when stimulus size changes, or the strength of associations might change in a more discrete fashion (i.e., associations switch at a particular size level). In Experiment 1, participants responded to stimulus color with a left/right keypress, and physical stimulus size had ten levels with 5 mm steps. Results showed correspondence effects for the smallest and the largest stimulus size only. In Experiment 2, physical stimulus size had six levels with 10 mm steps. Results showed (similar) correspondence effects for the smallest and some intermediate stimulus-size levels. In sum, the results point towards a discrete, or categorical, relationship between cognitive codes of stimulus size and left/right response codes. This pattern of results is consistent with an account of the correspondence effect in terms of the polarity correspondence principle.

## Introduction

The term ‘*stimulus-response compatibility*’ (SRC) refers to the fact that some mappings between a set of stimuli and a set of responses can produce faster and more accurate responses than other mappings (Proctor & Vu, 2006). The easier mapping is called a “compatible” condition, whereas the more difficult mapping is called an “incompatible” condition. In basic research, the effects induced by stimulus-response compatibility provide valuable insights into how features of stimulus and response are mentally represented. Moreover, by investigating compatibility effects, knowledge is gained about response selection, addressing the question how decisions between two competing response alternatives are made. For this purpose, compatibility effects including various dimensions of stimulus and response such as space and number have been investigated (Proctor & Vu, 2006).

One well-known example is the spatial compatibility effect (Fitts & Seeger, 1953). If the spatial locations of stimulus and response correspond, e.g., if a left response is required to a stimulus presented to the left of a reference object, responses are faster and more accurate in comparison to incompatible mappings, in which a left response is required to a stimulus presented to the right of the reference object. This effect emerges even if the stimulus’ spatial feature is entirely task-irrelevant as shown by the *Simon effect* (Hommel, 2011; Simon & Rudell, 1967).^1^

The so-called *SNARC effect* (Dehaene et al., 1990, 1993; Fias & Fischer, 2005), the spatial-numerical association of response codes, constitutes another instance of a compatibility effect, involving the spatial representation of numerical size. In the most common case of the SNARC effect, small numerical magnitudes are associated with the left side, whereas large numerical magnitudes are associated with the right side. This leads to a better performance for left-hand responses to small numbers and right-hand responses to large numbers. Similar to the Simon effect, the SNARC effect not only occurs for numerical size being task-relevant but also for numerical size being task-irrelevant (Fias et al., 2001; Yu et al., 2018). Moreover, the SNARC effect is independent from a particular response modality (e.g., Dehaene et al., 1993; Gevers & Lammertyn, 2005; Plaisier & Smeets, 2011) but the combination of responses from different modalities (e.g., hand and foot) may invert the effect (e.g., Hartmann et al., 2014). The most prominent explanation of the SNARC effect assumes a spatial cognitive representation of numerical magnitude, a so-called mental number line (e.g., Dehaene et al., 1993; Hubbard et al., 2005; Hartmann et al., 2011), but the origin and characteristics of this representation are still a matter of debate (e.g., Proctor & Cho, 2006; Shaki et al., 2012). Since the processing of numerical magnitude is at the core of the SNARC effect, one might assume that the size of the effect is related to mathematical ability, but studies exploring this relationship have produced rather mixed results (e.g. Cipora et al., 2015; Cipora et al., 2016; He et al., 2021).

Unlike the spatial compatibility effect and the SNARC effect, the compatibility effect between physical size and horizontally aligned response location has received only little research interest (Ren et al., 2011; Wühr & Seegelke, 2018). The compatibility effect between stimulus size and response location implies that left-hand responses are faster and more accurate to physically small stimuli, whereas right-hand responses are faster and more accurate to physically large stimuli (Wühr & Seegelke, 2018). The aim of the current research was to examine the nature of associations between stimulus size(s) and response location(s) that underlie the effect. Relatedly, we aimed at investigating the transition between stimuli perceived as “small”, thus associated with left-hand responses, and those perceived as “large”, thus associated with right-hand responses. Does the effect manifest itself as a categorical or a continuous relation? To address this question, we varied stimulus size on ten (Experiment 1) and six (Experiment 2) levels. Finally, the question arises whether the transition is located in the middle of the selected stimulus range or somewhere else.

### Compatibility between physical stimulus size and response location

Ren et al. (2011) were the first to demonstrate the effect by varying the size of a black circle on nine different levels. In a size-comparison task, participants had to indicate if the latter of two consecutively displayed circles was smaller or larger than the first one by responding with their left/right hand. With stimulus size being a task-relevant feature, a significant compatibility effect occurred for right-hand responses, which were faster to larger than to smaller circles, but not for left-hand responses. Wühr and Seegelke (2018) replicated the results and additionally observed the occurrence of the effect for left-hand responses, showing that right-hand responses were faster to large stimuli while left-hand responses were faster to small stimuli. Moreover, they expanded the evidence for the compatibility effect by demonstrating that the effect also emerges when physical size is task-irrelevant. Apart from varying stimulus size (small-large), Wühr and Seegelke (2018) also varied stimulus color (red-green), to which participants had to respond irrespectively of stimulus size with their left/right hand. Responses were faster in corresponding conditions (small-left; large-right) than in non-corresponding conditions (small-right; large-left), implying that physical size is processed automatically (see also Simmons et al., 2019 for similar results).

Two questions inevitably arise regarding the compatibility or correspondence effect between stimulus size and response location. Firstly, why are the two dimensions, namely stimulus size and response location, naturally associated and in which way are they interrelated? Accounts for explaining the intrinsic relationship between physical size and response location derive from *A Theory of Magnitude* (ATOM; Walsh, 2003) and the *polarity correspondence principle* (Proctor & Cho, 2006). In ATOM, Walsh (2003, 2015) assumes the existence of a generalized magnitude system, which uses the same metric for the representation and processing of space, time and quantity. Hence, the different domains are interrelated according to a monotonic mapping: “bigger, faster, brighter, further in one domain should correlate with bigger, faster, brighter, further in another” (Walsh, 2015, p. 557). Moreover, he assumes that the generalized magnitude system processes information about any size (cf. Walsh, 2003, p. 487), and thus ATOM provides an account for associations between physical size and response location. However, ATOM remains mute concerning particular patterns of associations between two dimensions.

Another account derives from the polarity correspondence principle (Proctor & Cho, 2006). According to this principle, in many binary decision tasks, a negative polarity is assigned to one stimulus and response alternative (e.g., small, left), whereas a positive polarity is assigned to the other stimulus and response alternative (e.g., large, right). These polarities are undeliberately processed, and a match between the polarities leads to faster and more accurate responses than a mismatch (see, also, Proctor & Xiong, 2015). Proctor and colleagues (2006, 2015) explain the SNARC effect by arguing that “small” (stimuli) and “left” (responses) are both assigned negative polarity, whereas “large” (stimuli) and “right” (responses) are assigned positive polarity. This assumption can be applied to the compatibility or correspondence effect between physical stimulus size and horizontal response location (Ren et al., 2011; Wühr & Seegelke, 2018). Contrary to ATOM, the polarity correspondence account implies that due to the binary classification of both dimensions into either “small” or “large” and either “right” or “left”, the association pattern should be categorical in nature.

The second question considering the correspondence effect arises as follows: Based on the associations between stimulus size and response location, how can an irrelevant stimulus feature activate a response alternative? *Dual-route models* have been put forward in order to account for effects of the irrelevant dimension in the spatial correspondence effect (Kornblum et al., 1990), the SNARC effect (Gevers et al., 2006) as well as the polarity correspondence principle (Proctor & Cho, 2006). Dual-route models assume two routes by which stimuli can activate responses. Through the conditional route, the correct response is selected to the relevant stimulus feature, based on the S-R assignments that are stored in working memory. At the same time, both relevant or irrelevant stimulus features can activate a response alternative based on long-term associations that are stored in long-term memory (cf. De Jong et al., 1994; Kornblum et al., 1990; Tagliabue et al., 2000). In corresponding conditions, the two routes will activate the same (i.e., correct) response, which will thus be quickly selected and executed. In non-corresponding conditions, however, the two routes will activate different responses, and the resulting response conflict will delay a response, due to additional processing for resolving the conflict, and sometimes even lead to an error. Applied to the correspondence effect between stimulus size and response location, the dual-route model can therefore explain the activation of an alternative response since task-irrelevant long-term associations between stimulus size and response location are processed simultaneously with task-relevant associations between stimulus color and response location in working memory.

### Possible relationships between physical size and horizontal location

**Figure 1:**
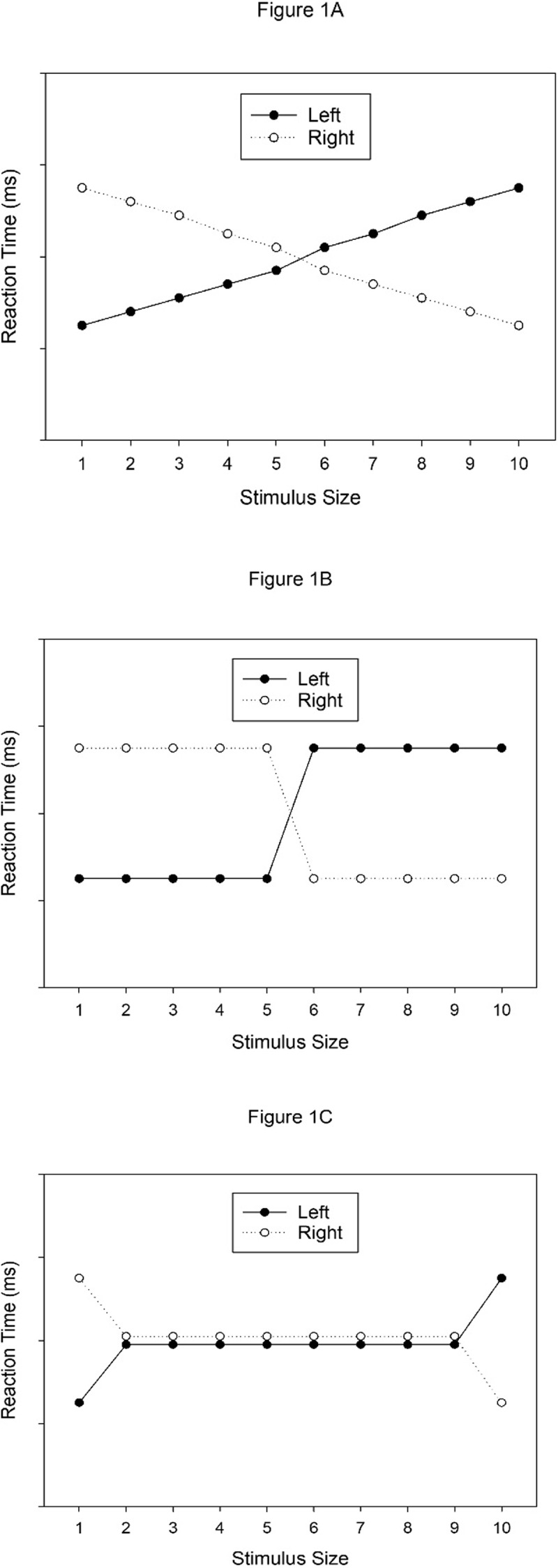
Different patterns of RT results for different patterns of associations between ten different stimulus-size codes and two response codes.

The main goal of this study is to explore in more detail the relationship between the dimension of physical (stimulus) size and horizontal (response) location, which leads to the compatibility or correspondence effects described in previous studies (e.g., Ren et al., 2011; Wühr & Seegelke, 2018). In particular, we would like to investigate how the individual manifestations of stimulus size, which are part of a stimulus set, are associated to the manifestations of the response, that is, the two response categories (left vs. right). Three cases, or types of relationship between stimuli and response categories, are conceivable. For all three cases, we assume that each individual stimulus has associations with both response codes (or both categories), but an increase of stimulus size leads to different changes in the strengths of associations in the three cases. For the following description of the three cases, we assume a stimulus set with ten stimuli of increasing size from one to ten size units. In the first case, the smallest stimulus has the strongest association (e.g., 10) with the left response, and the weakest association (e.g., 1) with the right response. With each individual increase of size, the association with the left response decreases by one unit (of association strength), while the association with the right response increases by one unit. Hence, a stimulus of intermediate size has an intermediate association strength (e.g., 5) to both the left and the right response, and the largest stimulus has the weakest association (i.e., 1) with the left response, and the strongest association (i.e., 10) with the right response. As a result, this pattern of associations would predict a gradually increasing RT function of left responses when stimulus size increases, and a gradually decreasing RT function for right responses when stimulus size increases (cf. ***Figure 1A***).

In the second case, the (five) smaller stimuli all have the same strong association (e.g., 10) with the left response, and the same weak association (e.g., 1) with the right response, whereas the opposite applies to the (five) larger stimuli. As a result, this pattern of associations would predict two step-shaped RT functions for left and right responses that cross at intermediate stimulus sizes (five or six). In particular, RTs of left responses are similarly short for the (four or five) smaller stimulus sizes, increase between the two intermediate sizes, and remain long for the (four or five) larger stimulus sizes. Conversely, RTs of right responses are similarly long for the (four or five) smaller stimulus sizes, decrease between two intermediate sizes, and remain short for the (four or five) larger stimulus sizes (cf. ***Figure 1B***).

Finally, in the third case, only the extreme-sized stimuli have different associations with the two response codes, whereas the remaining stimuli have similar associations with the response codes. In particular, the smallest stimulus would have a strong association (e.g., 10) with the left response, and a weak association (e.g., 1) with the right response. The largest stimulus would have a strong association with the right response and a weak association with the left response. All other stimuli would have intermediate associations of similar size (e.g., 5) with both responses. As a result, this pattern would predict RT differences between left and right responses for the two extreme stimulus sizes only, with left responses being faster than right responses to the smallest stimulus, right responses being faster than left responses to the largest stimulus, and similar RTs of both responses to the remaining stimuli (***Figure 1C***).

How do these three different patterns of associations fit with the two accounts for the origin of the compatibility effect described in the previous section? ATOM does not say much about the processing of the different, interconnected dimensions, and therefore seems to be consistent with all three possible scenarios. Things are different for polarity correspondence, however. In particular, one might envision two ways in which polarities could be assigned in tasks involving many stimuli (e.g., ten sizes) and two responses. If a task involves ten stimuli which vary on a metric dimension such as size, it seems possible that polarities are assigned to the two extreme stimulus values. Hence, the smallest stimulus would receive a negative polarity, the largest stimulus would receive a positive polarity, whereas intermediate stimulus values would not receive any polarity. The result would be a pattern as in ***Figure 1C***. Alternatively, it seems possible that the stimulus set is divided into two (more or less) equally large subsets of five stimuli each, and all stimuli in the subset of smaller stimuli receive negative polarity, whereas all stimuli in the subset of larger stimuli receive positive polarity. The result of this assignment would be a pattern as in ***Figure 1B***. Hence, with some auxiliary assumptions, the polarity correspondence principle seems consistent with patterns 1B and 1C, but not with pattern 1A.

### Relationship between numerical size and response location

While the shape of the compatibility or correspondence effect between physical stimulus size and response location has not yet been explored, the issue has been addressed with numerical stimulus sizes, that is, with regard to the SNARC effect. Interestingly, concerning the SNARC effect, the function relating numerical size to effect size (i.e., RT of right response minus RT of left response) appears to be task-specific. In particular, this function typically has a continuous shape, consistent with pattern 1A (e.g., Dehaene et al., 1993; Fias et al., 1996), except for tasks requiring the explicit classification or comparison of magnitudes. In the latter case, the function relating numerical size to effect size has a categorical, step-like shape, consistent with pattern 1B (e.g., Gevers et al., 2006; Shaki & Petrusic, 2005; see Wood et al., 2008, for a meta-analysis). Gevers et al. (2006) explain the effect’s categorical shape in magnitude classification tasks as a result of two factors: An increase of RTs when the distance between the to-be-compared numbers (i.e., test stimulus and standard) decreases, a phenomenon called “distance effect” (e.g., Moyer & Landauer, 1967), and an increase of the size of the SNARC effect with increasing RT. Together, these two factors turn the continuous shape of the effect-size function into a categorical, step-like function in magnitude classification tasks (Gevers et al., 2006).

It might be tempting to assume that the function relating physical stimulus size to the size of the correspondence effect looks similar to the function for the SNARC effect. That is, one might expect to find a continuous effect size function (e.g., ***Figure 1A***), as for the SNARC effect, in tasks that do not require the explicit classification or comparison of magnitudes (cf. Wood et al., 2008). However, several differences between numerical and physical magnitude could also lead to different effect-size functions. Firstly, numerical size constitutes a discrete variable whereas physical size constitutes a continuous variable. Crucially, it has been proposed that the processing of magnitudes might not involve one shared approximate number system (ANS) but instead separate yet interactive systems for the processing of discrete and continuous quantities (Leibovich & Henik, 2014). Secondly, there are well established and overlearned instances of numerical size, which are therefore easily recognized and discriminated in each situation including psychological experiments. In contrast, well established and overlearned instances of physical size do not exist, and therefore the particular instances of physical size have to be learned anew in each situation including our experiments (cf. Nys & Content, 2012).

### The present study

The goal of this study is to explore the nature of the associations between physical stimulus size and left-right response codes, which lead to the compatibility or correspondence effects described in previous studies (e.g., Ren et al., 2011; Wühr & Seegelke, 2018). Therefore, we investigated the shape of the functions relating physical stimulus size to the latencies of left and right responses to these stimuli. The shape of these functions is – in turn – not only informative about the relative strengths of associations between the cognitive representations of stimulus sizes, on the one hand, and the (response) categories “left” and “right”, on the other hand, but it is also informative about the applicability of the polarity correspondence and the ATOM account. In two experiments, we used a Simon-like task in which participants responded to the color (red or green) of a filled square by pressing a left key with the left hand, or a right key with the right hand. In contrast to our previous experiments (e.g., Wühr & Seegelke, 2018), however, we used more than two values of stimulus size in the present experiments. In particular, there were ten stimulus sizes in Experiment 1 and six stimulus sizes in Experiment 2.

## Experiment 1

In Experiment 1, participants had to perform a color discrimination task by responding to the color (red or green) of a filled square with their left/right hand. Additionally, stimulus size was varied as an irrelevant feature between ten different sizes, thus leading to corresponding and non-corresponding conditions. To our knowledge it is the first study to investigate the shape of the correspondence effect with a continuous quantity, revealing insights into the relative strength of the automatic associations between different manifestations of physical size and response location as stored in long-term memory.

Similar to our previous experiments, we additionally employed a size-comparison task, in which participants were asked to distinguish between two adjacent stimulus sizes by responding to the larger one. Being particularly relevant when stimulus size varies in small steps, this task ensured that participants were able to reliably differentiate between adjacent sizes. Moreover, the task controlled for the potential ambiguity that the differences between imperative stimuli might be perceived as variations in distance instead of variations in size. For this reason, task order of the size-comparison and the color-discrimination task was varied between participants. If participants intuitively perceive the variation of the irrelevant stimulus feature as variation in size, task order should not have any effect on the correspondence effect. However, if the irrelevant stimulus feature is ambiguous for participants, we expect the correspondence effect to be larger for the group that performed the size-comparison task first (Wühr & Seegelke, 2018).

### Methods

#### Participants

In our previous study, the correspondence effect was quite strong (i.e., \eta _p^2 = .36) in a sample of 40 participants (Wühr & Seegelke, 2018). The present experiment, however, had a different design, and we were mainly interested in a two-way (2 × 10) interaction. Lacking studies for conducting an informed power analysis, we chose a sample size of 48 participants, which gives high power (i.e., 1–*β* = .95) for detecting an interaction effect of intermediate size ( \eta _p^2 = .12 = .36/3).

All experiments reported in this manuscript had been approved by the Research Ethics Committee at TU Dortmund University (approval no. 2018–09). Participants were 48 volunteer students (32 female, 16 male) with a mean age of 22 years (range 18–28 years), who received course credit in exchange. According to self-report, all participants were right-handed and had normal or corrected to normal vision. All participants gave their informed consent prior to participation.

#### Apparatus and stimuli

At a distance of approximately 50 cm, participants sat in front of a 17-inch color monitor. Stimulus presentation and measurement of the key-press responses (response time and error percentage) were ensured by means of the software E-Prime running on a customary computer. At the beginning of each trial, a small plus sign (Courier font, font size 18) presented at the screen center served as a fixation point. Serving as imperative stimuli, ten squares of different sizes varying between 5 mm and 50 mm with increments of 5 mm respectively were presented in red or green color on a gray background (E-Prime color “silver”) at the center of the screen. Participants responded by pressing the left Control key or the right Enter key with the index fingers of their left and right hand, respectively. The center of the keyboard was aligned to the body midline and fixed to the table. The two relevant keys were marked with black tape.

#### Procedure

The experiment consisted of two tasks, one size-discrimination task as well as one color-discrimination task. The order of both tasks was counterbalanced across participants and randomly assigned. Instructions at the beginning of each task informed participants about the content and the procedure of the task.

In the size-discrimination task, participants were shown two squares of adjacent sizes, which were placed next to each other at the screen center. In each trial, participants had to respond to stimulus size by pressing the key on the side of the larger square. The task consisted of one experimental block with 36 trials (9 pairs × 2 colors × 2 spatial arrangements) presented in random order. In each experimental trial, the fixation point was displayed for 500 ms, followed by the presentation of the stimulus array until a response was made or for a maximum period of 2000 ms. In case of an incorrect or lacking response, a corresponding error message was shown for 1000 ms.

In the color-discrimination task, participants responded to the color of the square by pressing the left or right key. This task included 11 blocks, one practice block containing 20 trials (10 sizes × 2 colors) and ten experimental blocks containing 40 trials (10 sizes × 2 colors presented twice). Each trial started with the presentation of the fixation point for 400 or 600 ms; the two intervals occurred with equal frequency in each condition. The stimulus was displayed until a keypress was made or for a maximum of 2000 ms. A corresponding error message was shown for 1000 ms if an incorrect or no key press was made. Participants were able to optionally take a break between the blocks or start the subsequent one.

The experiment took about 30 minutes. During the size-discrimination task and the practice block of the color-discrimination task, the experimenter stayed in the laboratory. The experimenter left the room for the experimental blocks of the color-discrimination task.

#### Design

The color-discrimination task had a 10 (Stimulus Size) × 2 (Response Location) within-subjects design, which resulted in 20 distinct conditions. The factor stimulus size had ten levels; the factor response location had two levels (i.e., left vs. right). The order in which the size-discrimination and the color-discrimination task were executed was manipulated as a between-subjects variable. The dependent variables were reaction times (RT) of correct responses, and the percentages of errors (PE). For each participant, we computed RT and PE means in every condition. Trials with RT below 100 ms or above 1,500 ms were excluded.

In this study, we were particularly interested in the specific form of the two-way interaction of stimulus size and response location. To properly analyze the form of this interaction, and to determine the cutting point of the functions relating stimulus size and RT for each response, it is necessary to remove the impact of a main effect of response location if it occurred (e.g., Harwell, 1998; Rosnow & Rosenthal, 1989). Moreover, for analyzing the form of the two-way interaction, we also planned a set of comparisons between RTs (and error percentages) for left and right responses at each level of stimulus size. We used uncorrected *t* tests for these comparisons because these tests were planned a-priori on theoretical grounds. Moreover, these *t* tests were done one-tailed for the extreme sizes because all three patterns of associations predict superior performance of left responses for the smallest stimulus sizes (i.e., level 1), and superior performance of right responses for the largest stimulus sizes (i.e., level 10). For the intermediate stimulus sizes, the different patterns of associations make divergent predictions, hence these *t* tests were done two-tailed.

### Results

#### RTs in color-discrimination task

A preliminary mixed-design analysis of variance (ANOVA) with the between-subject factor *Task Order* (size-discrimination task – color-discrimination task; color-discrimination task – size-discrimination task) as well as both within-subjects variables *Stimulus Size* (small – large on ten increments) and *Response Location* (left vs. right) did not reveal any effect of *Task Order*. The between-subject factor was therefore dropped from further analysis. A two-factorial repeated measures ANOVA was conducted with *Stimulus Size* and *Response Location* as independent variables, and RT as dependent variable.

A significant main effect of *Stimulus Size, F*(7.05, 331.28) = 11.26, *MSE* = 532.55, *p* < .001, \eta _p^2 = .193, reflected shorter RT for larger squares (level 10: *M* = 373.72 ms, *SE* = 6.87) than for smaller squares (level 1: *M* = 390.85 ms, *SE* = 6.35) (a Greenhouse-Geisser-correction was conducted as sphericity was violated). The main effect of *Response Location* was also significant, *F*(1, 47) = 22.20, *MSE* = 1356.93, *p* < .001, \eta _p^2 = .321, revealing that right-hand responses were faster (*M* = 367.68 ms, *SE* = 6.14) than left-hand responses (*M* = 378.89 ms, *SE* = 6.21). The most important finding, however, was the significant *Stimulus Size × Response Location* interaction, *F*(9, 423) = 3.60, *MSE* = 391.11, *p* < .001, \eta _p^2 = .071.

**Figure 2:**
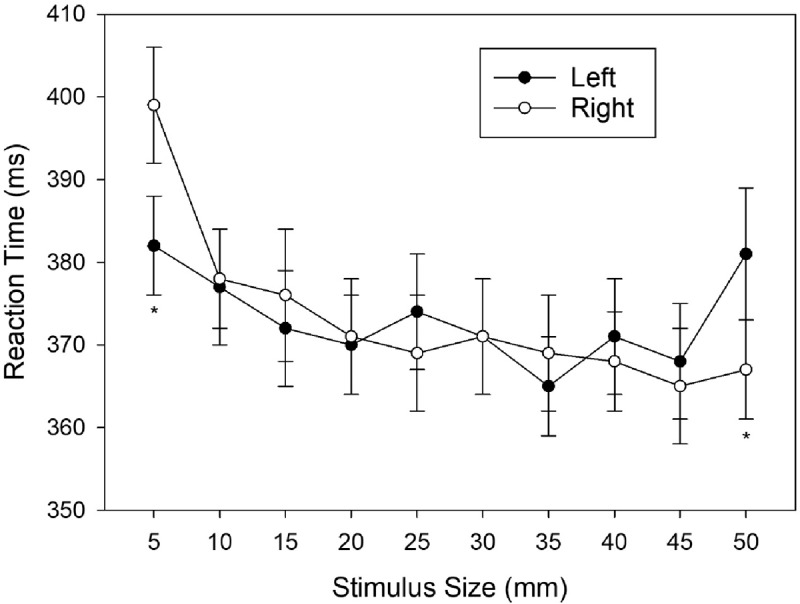
Mean Reaction Times (ms) observed in Experiment 1 as a function of Stimulus Size and Response (main effect of response removed from data). Error bars represent standard errors (between participants). An asterisk indicates a significant difference at this particular level of stimulus size.

To meet the purpose of our study, it was necessary to remove the (significant) main effect of response location from the data (Harwell, 1998; Rosnow & Rosenthal, 1989). The following analyses are based on the adjusted data. The adjusted cell means are depicted in ***Figure 2***. To determine the form of the interaction effect, we compared RTs for left and right responses at each level of the factor stimulus size by planned comparisons (i.e., *t* tests). A Shapiro-Wilk test showed that the distribution of pair-wise differences was normal for 9 out of 10 differences (the only exception was size level 5), hence we decided to use a parametric test. The set of tests revealed a significant advantage for left-hand responses to the smallest stimulus size (5 mm), *t*(47) = –4.28, *p* < .001, *d*_z_ = –.618, and a significant advantage for the right-response to the largest stimulus size (50 mm), *t*(47) = 2.25, *p* = .014, *d*_z_ = .325. The remaining tests were not significant, all *t*s(47) < 1.3, all *p*s > .20.

#### Error percentages in color-discrimination task

The error percentages from the color-discrimination task were subjected to a two-factorial ANOVA with *Stimulus Size* and *Response Location* as independent variables. The results were similar to the RT results. The main effect of *Stimulus Size, F*(6.85, 321.99) = 2.152, *MSE* = 15.004, *p* = .039, \eta _p^2 = .044, and the main effect of *Response Location, F*(1, 47) = 7.474, *MSE* = 15.460, *p* = .009, \eta _p^2 = .137, were significant. The main effect of *Stimulus Size* indicated a decrease of errors when stimulus size increased. The main effect of *Response Location* refers to a higher error percentage with right responses (*M* = 2.918, *SD* = 4.745) than with left responses (*M* = 2.225, *SD* = 3.785) and is in line with our observation that right responses are faster than left responses and consequently lead to more erroneous responses.

Again, the most important finding was a significant two-way interaction between *Stimulus Size* and *Response Location, F*(6.41, 301.23) = 3.693, *MSE* = 21.516, *p* = .001, \eta _p^2 = .073. The non-adjusted cell means are shown in ***Table 1***. We, however, refrained from further analyzing the error percentages for the following reasons. A Shapiro-Wilk test revealed that the pairwise differences were significantly different from normal for each size level, indicating the use of a non-parametric test, such as the Wilcoxon signed-rank test, for performing pair-wise comparisons. However, the most frequent observation was zero errors both with left responses (69%) and with right responses (63%), contributing to a large number of ties (i.e., 48% of the pairs), which would have compromised the results of the non-parametric test.

**Table 1:** Mean error percentages observed in Experiment 1 as a function of Stimulus Size and the required Response (R).


	STIMULUS SIZE									

	1	2	3	4	5	6	7	8	9	10

Left R	1.958	1.190	1.994	2.402	2.658	1.971	3.056	2.110	2.954	1.953

Right R	5.196	4.100	2.957	3.963	1.982	1.954	2.166	3.179	1.803	1.884

Difference	–3,238	–2,910	–0,963	–1,561	0,676	0,017	0,890	–1,069	1,151	0,069


#### Size-discrimination task

Mean RT in the size-discrimination task was 445 ms (*SD* = 61), and mean error percentage was 2.546 (*SD* = 4.432). The low error percentage indicates that when directly comparing stimulus sizes, participants were able to reliably distinguish between adjacent sizes, even if stimulus size varies in small steps.

### Discussion

Since task order did not exert any significant influence, it can be concluded that size differences were interpreted as such and not as variations in distance. Moreover, participants were able to reliably discriminate between relative physical sizes. A correspondence effect between physical stimulus size and horizontal response location was found even though stimulus size was task-irrelevant, suggesting that physical stimulus size was undeliberately associated with left-/right-hand responses. However, the correspondence effect in Experiment 1, involving ten different stimulus sizes, was restricted to the two most extreme stimulus-size values. The left response was faster than the right response to the smallest stimulus (5 mm), and the right response was faster than the left response to the largest stimulus (50 mm), whereas no reliable correspondence effects occurred for the eight intermediate stimulus values. Hence, the pattern observed in Experiment 1 resembled the pattern shown in ***Figure 1C***.

## Experiment 2

The main purpose of Experiment 2 was to explore whether and how a change in the stimulus set would affect the shape of the stimulus size – response location correspondence effect. A possible – post-hoc – account for the finding of Experiment 1, where only the most extreme stimulus values were able to produce significant correspondence effects, might be that the most extreme stimulus values are more distinct, or salient, than the other levels of stimulus size. In particular, the large number of stimulus sizes (i.e., more than seven; Miller, 1956) and the small differences between adjacent stimulus sizes might have rendered the recognition of individual stimuli very difficult, and impeded learning the ordinal sequence of stimulus sizes in the stimulus set (cf. Ashby & Ell, 2001 for evidence that the number of stimuli per category affects classification learning). Therefore, we tried to increase the recognition of individual stimuli, and foster the learning of the ordinal stimulus sequence, by (a) reducing the number of stimulus sizes from ten to six, and (b) by increasing the steps between adjacent stimulus sizes from 5 mm to 10 mm.

If the associations between individual stimulus sizes and response codes were rather hard-wired, Experiment 2 should produce a very similar pattern of results as Experiment 1. In particular, in that case we should observe correspondence effects for only the two most extreme values of stimulus size, which are very similar to the two most extreme values from Experiment 1. If, however, the size of the stimulus set, and the recognizability of individual stimuli can modulate the pattern of correspondence effects, a different pattern might emerge in Experiment 2. In particular, in the latter case, the pattern might turn from the one shown in ***Figure 1A*** (and observed in Experiment 1) to one of the patterns shown in ***Figure 1B*** or in ***Figure 1C***. Such a finding would show that the pattern of associations between individual stimulus sizes and response codes are not hard-wired and immutable but are malleable by task variables.

### Methods

#### Participants

58 volunteer students (51 female, 7 male) with a mean age of 22 years (range 19–38 years) participated in Experiment 2. According to the *Edinburgh Handedness Inventory* (Oldfield, 1971), all participants were right-handed (EHI score: *M* = 71.81; *SD* = 14.53; range = 40–100) and had normal or corrected to normal vision. Prior to participation, the students gave their informed consent. They received course credit in exchange.

#### Apparatus and stimuli

We used the same apparatus and stimuli as in Experiment 1, with the following exceptions: Instead of ten different sizes ranging between 5 mm and 50 mm (side length) with increments of 5 mm each, we now used six different sizes ranging between 10 mm and 60 mm (side length) with increments of 10 mm each.

#### Procedure

The procedure employed in Experiment 2 was also equivalent to the one in Experiment 1 except for the reduced number of six different stimulus sizes. In the size-discrimination task, the combination of 5 pairs × 2 colors × 2 arrays resulted in 20 trials. In the color-discrimination task, the practice block contained 12 trials (6 sizes × 2 colors presented once), whereas each of the ten experimental blocks contained 24 trials (6 sizes × 2 colors presented twice).

#### Design

The design of the color-discrimination task was a 6 (Stimulus Size) × 2 (Response Location) within-subjects design, resulting in 12 different conditions. The independent variable *Stimulus Size* had six levels (see Method section). The independent variable *Response Location* had two levels (left vs. right). The order in which the size- and color-discrimination task were performed was varied between participants. Dependent variables were RTs of correct responses and the percentages of errors (PE). In all other aspects, our strategy for data analysis was similar to that in Experiment 1.

### Results

#### RTs in color-discrimination task

**Figure 3:**
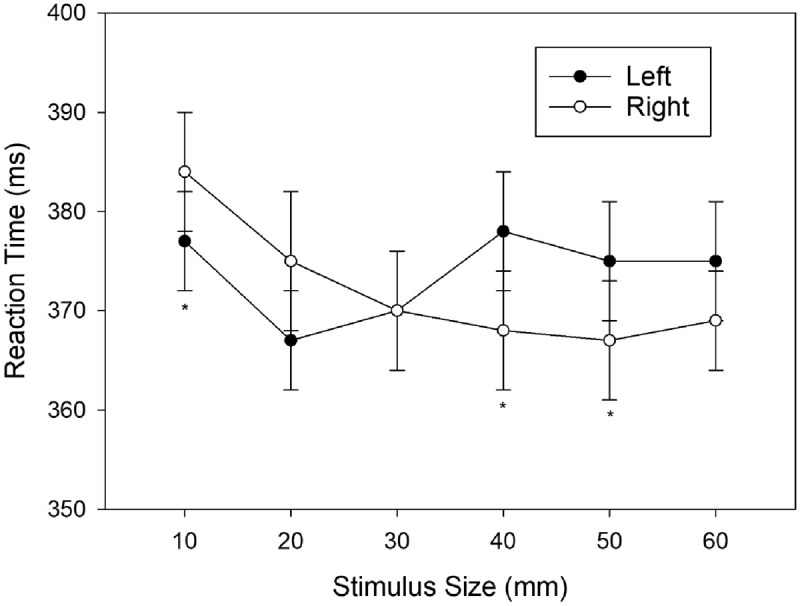
Mean Reaction Times (ms) observed in Experiment 2 as a function of Stimulus Size and Response. Error bars represent standard errors (between participants). An asterisk indicates a significant difference at this particular level of stimulus size.

A preliminary mixed-design ANOVA with the between-subject variable *Task Order* and both within-subject variables *Stimulus Size* and *Response Location* failed to reveal a significant effect of *Task Order*, which was thus excluded from further analysis. A two-factorial repeated measures ANOVA was conducted with *Stimulus Size* and *Response Location* as independent variables and reaction time as dependent variable. A significant main effect of *Stimulus Size, F*(5, 285) = 4.15, *MSE* = 422.17, *p* = .001, \eta _p^2 = .068, reflected shorter RTs for larger stimuli (level 6: *M* = 372.02 ms, *SE* = 5.27) in comparison to smaller stimuli (level 1: *M* = 380.43 ms, *SE* = 5.27). The main effect of *Response Location* did not reach significance, *F*(1, 57) = 0.34, *MSE* = 846.76, *p* = .564, \eta _p^2 = .006. The most important result was the two-way interaction between *Stimulus Size* and *Response Location, F*(5, 285) = 3.82, *MSE* = 399.13, *p* = .002, \eta _p^2 = .063. The cell means are shown in ***Figure 3***.

To determine the form of the interaction effect, we compared RTs for left and right responses at each level of the factor stimulus size by planned comparisons. A Shapiro-Wilk test showed that the distribution of pair-wise differences was significantly different from normal for sizes 30 mm, 40 mm and 50 mm; hence we decided to use the non-parametric Wilcoxon test for this set of comparisons. These comparisons revealed shorter RTs for left responses as compared to right responses for stimulus size 10 mm, *W*(57) = 634, *p* = .044, *r* = –.259, whereas RT were not different for stimulus size 20 mm, *W*(57) = 676, *p* = .166, *r* = –.210, and stimulus size 30 mm, *W*(57) = 936, *p* = .536, *r* = .094. In contrast, RTs were shorter for right responses as compared to left responses for stimulus size 40 mm, *W*(57) = 1,207, *p* = .007, *r* = .411, and 50 mm, *W*(57) = 1,136, *p* = .030, *r* = .328, but not for stimulus size 60 mm, *W*(57) = 1016, *p* = .108, *r* = .188.

#### Error percentages in color-discrimination task

The error percentages from the color-discrimination task were subjected to a two-factorial ANOVA with *Stimulus Size* and *Response Location* as independent variables. The main effect of *Stimulus Size, F*(4.11, 234.41) = 0.863, *MSE* = 12.148, *p* = .489, \eta _p^2 = .015, was not significant. However, the main effect of *Response Location* was significant, *F*(1, 57) = 4.618, *MSE* = 12.444, *p* = .036, \eta _p^2 = .075, indicating a lower error percentage when a left response was required (*M* = 1.976, *SD* = 3.247) than when a right response was required (*M* = 2.550, *SD* = 3.993). Finally, the interaction between *Stimulus Size* and *Response Location, F*(5, 285) = 3.381, *MSE* = 9.651, *p* = .006, \eta _p^2 = .056, was also significant. The non-adjusted cell means are shown in ***Table 2***. However, we refrained from further analyzing the error percentages for the same reasons that already applied to Experiment 1. First, a Shapiro-Wilk test revealed that the pairwise differences were significantly different from normal for each size level, indicating the use of a non-parametric test, such as the Wilcoxon signed-rank test, for doing pair-wise comparisons. However, as in Experiment 1, the most frequent observation was zero errors both with left responses (66%) and with right responses (61%). This result contributed to a large number of ties (i.e., 59% of the pairs), which would have compromised the results of the non-parametric test.

**Table 2:** Mean error percentages observed in Experiment 2 as a function of Stimulus Size and the required Response (R).


	STIMULUS SIZE					

	1	2	3	4	5	6

Left R	1.437	1.868	2.155	2.083	2.371	1.940

Right R	4.023	2.155	2.730	2.083	1.868	2.443

Difference (left–right)	–2.586	–0.287	–0.575	0	0.503	–0.503


#### Size-discrimination task

Mean RT in the size-discrimination task was 424 ms (*SD* = 58), and mean error percentage was 1.121 (*SD* = 2.302). Pairwise comparisons showed that size discrimination was both faster, *t*(104) = 1.836, *p* = .035, *d*_z_ = 0.358, and more accurate, *t*(104) = 2.128, *p* = .018, *d*_z_ = 0.415, in Experiment 2 than in Experiment 1. We used one-tailed tests because we expected better performance in Experiment 2 than in Experiment 1 due to the larger size steps in Experiment 2.

### Discussion

Again, a correspondence effect between physical stimulus size and horizontal response location was found, indicating the robustness of associations between physical size and left/right response codes. In particular, left responses were faster than right responses for the smallest size-level (10 mm), and right responses were faster than left responses for two out of three larger size-levels (i.e., 40, and 50 mm). Hence, once more, the results indicate that smaller sizes are more strongly associated with the left response than the right response, whereas the opposite is true for the larger stimulus sizes. Results of Experiment 2 revealed three noteworthy differences with regard to those of Experiment 1. Firstly, in contrast to Experiment 1, correspondence effects in Experiment 2 were not only significant for extreme size levels, but also significant for intermediate size-levels. Secondly, correspondence effects remained relatively constant both below and above the intersection point at size-level three. Hence, whereas the results of Experiment 1 matched the pattern of ***Figure 1C***, the results of Experiment 2 seem to match the step-shaped pattern of ***Figure 1B***. Thirdly, a comparison of performance in the size-discrimination task between Experiments 1 and 2 revealed that size-discrimination was easier in Experiment 2.

## General Discussion

In the present study, we conducted two experiments with more than two levels of stimulus size in order to explore the nature of associations between physical stimulus-size codes and left/right response codes, which are responsible for compatibility and correspondence effects between physical stimulus size and left/right responses. In Experiment 1, participants responded to stimulus color with a left or right keypress, and physical stimulus size was varied on ten levels. Results showed a correspondence effect for the smallest stimulus size, for which the left response was faster than the right one, and the largest stimulus size, for which the right response was faster than the left one. No correspondence effects occurred for intermediate levels of physical stimulus size. In Experiment 2, physical stimulus size was varied on six levels. Results showed correspondence effects for intermediate levels with faster left-hand than right-hand responses for stimulus-size levels smaller than 30 mm, and faster right-hand than left-hand responses for stimulus-size levels larger than 30 mm. In sum, the results point towards a discrete, or categorical, relationship between cognitive codes of stimulus size and left/right response codes.

### Possible relationships between physical size and horizontal location

The results of our experiments, and the shape of the size-RT functions for the two responses are consistent with the polarity correspondence principle if some auxiliary assumptions are made. This principle posits that, in many binary decision tasks, observers undeliberately assign negative polarity to one stimulus and response alternative, and positive polarity to the other stimulus and response alternative (Proctor & Cho, 2006; Proctor & Xiong, 2015). This should primarily happen if salient features of stimuli and responses may be considered as two opposing poles, such as small versus large, or left versus right. The correspondence or non-correspondence of stimulus and response then facilitates or impedes the selection and execution of the correct response to a given stimulus. In particular, matching polarities (e.g., when a small S requires a left R, or a large S requires a right R) facilitate response selection, whereas mismatching polarities (e.g., when a small S requires a right R, or a large S requires a left R) impede response selection, producing a compatibility (or correspondence) effect.

Although the polarity correspondence principle was originally proposed for binary-decision tasks only, there are two ways in which the principle can be extended to tasks with more than two stimulus values. In tasks with many stimuli (e.g., seven or more; e.g., Ashby & Ell, 2001; Miller, 1956) varying on a metric dimension such as size, it may be difficult to learn the ordinal position of each stimulus, but it may be possible to recognize the two most extreme stimulus values, that is, the smallest and the largest stimulus.^2^ As a result, in such a task, opposing polarities may be assigned to the extreme stimulus values but not to intermediate stimulus values. The result would be a pattern as in ***Figure 1C***, which we observed in Experiment 1. Alternatively, in tasks with a moderate number of stimuli (e.g., 3–7), it may be possible to learn the ordinal position of each stimulus on the critical stimulus dimension, even if this was irrelevant for the task. As a result, the stimulus set could be divided into two subsets of stimuli, and all stimuli in the subset of smaller stimuli receive negative polarity, whereas all stimuli in the subset of larger stimuli receive positive polarity. The result of this assignment would be a pattern as in ***Figure 1B***, which we observed in Experiment 2.

### A comparison to the SNARC effect

The results of our two experiments appear least consistent with the notion of gradually increasing and decreasing association strengths between representations of physical stimulus size and left/right response codes with increasing stimulus size. This would have produced a pattern as shown in ***Figure 1A***, which was not observed here. The pattern of gradually changing size-RT functions for left and right responses would have been expected if the associations between representations of physical stimulus size and left/right response codes are similar to the associations between numerical size and left/right response codes, which produce the SNARC effect.

In studies on the SNARC effect, gradually changing size-RT functions were observed when numerical size was irrelevant for the task at hand (e.g., Dehaene et al., 1993; Fias et al., 1996). In contrast, step-shaped size-RT functions, similar to the pattern in ***Figure 1B***, were observed when numerical size was relevant for the task at hand (e.g., Gevers et al., 2006; Shaki & Petrusic, 2005). The change of the size-RT functions from gradual to step-shaped in the latter case was explained by the combined influence of the so-called distance effect, and an increase of the size of the SNARC effect with increasing RTs (e.g., Gevers et al., 2006; Wood et al., 2008).

Most importantly, physical stimulus size was task-irrelevant in our experiments. Thus, if the associations between physical stimulus size and left/right response codes were similar to the associations between numerical stimulus size and left/right response codes, and if these associations were affected by similar mechanisms, we should have observed the gradual pattern of size-RT functions (as in ***Figure 1A***). The fact that we did not observe this pattern, hence, implies that the associations between physical stimulus size and left/right response codes are different from the associations underlying the SNARC effect, and/or that these associations are affected by different mechanisms. Future research may further address these implications.

### Alternative accounts?

As an alternative account ATOM, however, still cannot be ruled out. By characterizing the relationship between different quantities and space as being monotonous in nature, Walsh (2003, 2015) merely predicts unidirectional associations between them, i.e. an increasing × should be associated with an increasing or at least constant y. However, a monotonous relationship does not prescribe continuousness, neither does is preclude a categorical pattern of associations nor make any specific predictions about association patterns in general. All of the three afore-mentioned patterns which map physical size onto space (***Figure 1***) including the patterns we observed in Experiment 1 and Experiment 2 are monotonous in nature and therefore consistent with ATOM. Nevertheless, the fact that the correspondence effect between stimulus size and response location on the one hand and the SNARC effect on the other hand seem to rely on different association patterns weakens ATOM as a potential candidate to account for the effect. In particular, this finding challenges the assumption of ATOM, that one generalized magnitude system exists which uses the same metric for the processing of different quantities, space and time (Walsh, 2003, 2015).

### Task-specific versus task-unspecific associations

Although the present experiments were not primarily designed to address this issue, the results provide some clues as to whether the associations between physical stimulus size and left/right response codes are hard-wired, and thus immune to changes in task context, or malleable, and thus sensitive to changes in task context. Two results of our experiments are informative here. The first finding arises from comparing the present results, obtained with large (non-binary) stimulus sets, to the results of previous experiments, obtained with small (binary) stimulus sets. In previous studies, we always used a small stimulus of 20 mm side length, and a large stimulus of 40 mm side length (e.g., Wühr & Seegelke, 2018; Seegelke & Wühr, 2018). These conditions produced reliable correspondence effects, where the left response was faster and more accurate than the right response to the small stimulus, and the right response was faster and more accurate than the left response to the large stimulus. These two stimulus-size levels were also included in the larger stimulus sets of the present experiments. However, in Experiment 1, we neither observed a correspondence effect for the 20 mm stimulus nor for the 40 mm stimulus.

The second finding arises from comparing the results of Experiments 1 and 2, which produced different patterns of correspondence effects with different sets of stimulus sizes. Together, the two findings disprove the idea that the associations between a particular physical stimulus size and the left/right response codes are hard-wired, and immune to task context. Rather, the results suggest that stimuli varying in physical size are classified as “small” or “large” with regard to the range of stimulus sizes in the current stimulus set. The classification process may result in a stimulus being tagged as “small” or “large”, and these tags may then activate (possibly pre-existing) associations between “small” and “left” (responses), and between “large” and “right” (responses), respectively.

### Limitations and future research

In both experiments an unexpected main effect of stimulus size occurred, in that RTs decreased when stimulus size increased. This result implies that color discrimination was more difficult for smaller than for larger stimuli. The main effect of stimulus size is not desirable because we cannot exclude that variations in the difficulty of color discrimination with size also affect the magnitude of the correspondence effects at particular size levels. We suggest avoiding main effects of stimulus size in future experiments, for example, by choosing relevant stimulus features other than color. In a more recent study, we used stimulus shape (i.e., circle and square) as the relevant stimulus feature, and did not observe a main effect of stimulus size in this experiment (Wühr & Richter, 2021, Experiment 2).

According to the polarity correspondence principle, correspondence effects occur when two concepts share the same polarity they were assigned. Proctor and Cho (2006) assume that space is coded verbally into the two opposing concepts “left” and “right”, whereas quantity is coded verbally into the opposing poles “small” and “large” (see also Gevers et al., 2006). Gevers et al. (2010) accordingly term the polarity correspondence principle the verbal-spatial account. Contrary to the verbal-spatial account, the so-called visuo-spatial account proposes “a tight correspondence between the position of a [quantity] on a continuous left-to-right-oriented representational medium […] and the spatial position of the response” (Gevers et al., 2010, p. 181). The mental number line constitutes an instance of such a visuo-spatial account in order to explain the SNARC effect.

In our study, there is a confound between the verbal-spatial and the visuo-spatial coding system. Faster and more accurate left-hand responses to small stimuli could occur “either because the response is located left in physical space (visuo-spatial coding) or because the label small associated to the small [size] evokes the concept ‘left’ (verbal-spatial coding)” (Gevers et al., 2010, p. 182). Our study therefore does not allow to reliably distinguish between a verbal-spatial or a visuo-spatial account. Gevers et al. (2010) addressed this issue by eliminating the physical lateralization in the response medium. Instead of making left/right key presses, participants had to verbally respond by saying “left” or “right”. In this experimental design, only verbal-spatial coding may occur but no visuo-spatial coding, which allows to distinguish between both coding systems. Future research should investigate if the correspondence effect between physical stimulus size and left/right response still occurs even if no visuo-spatial coding takes place. We have already begun to address this issue empirically.

## Conclusion

The results of our experiments suggest a discrete, or categorical, relationship between cognitive codes of stimulus size and left/right response codes, which underlie compatibility or correspondence effects between physical stimulus size and left/right responses. Moreover, the results also suggest that only extreme stimulus values are (inadvertently) classified as small or large when the number of stimuli is high (i.e., 10, Experiment 1), whereas also intermediate stimulus values are classified when the number of stimuli is smaller (i.e., 6, Experiment 2). The pattern of results is consistent with the polarity correspondence principle, which assumes that negative and positive polarities are assigned to the members of binary stimulus and response categories, and the correspondence or non-correspondence of stimulus and response polarities in a given trial affects performance.

## Data Accessibility Statement

The data sets obtained in Experiments 1 and 2 can be accessed from a public repository called “SowiDataNet|datorium” (*https://doi.org/10.7802/2348*).

## Footnotes

We use the term ‘compatibility effect’ to denote the effect of different mappings between relevant stimulus and relevant response features. In contrast, we use the term ‘correspondence effect’ to describe the effect of different combinations between irrelevant stimulus features and relevant response features.

A corresponding observation, called the “edge effect”, is known from research on absolute identification performance (e.g., Weber et al., 1977).

## Author Information

Melanie Richter, Technische Universität Dortmund, Institut für Psychologie, Emil-Figge-Strasse 50, D-44227 Dortmund, Germany (melanie2.richter@tu-dortmund.de). Peter Wühr, Technische Universität Dortmund, Institut für Psychologie, Emil-Figge-Strasse 50, D-44227 Dortmund, Germany (*peter.wuehr@tu-dortmund.de*).
